# The efficacy and safety of androgen analog oxandrolone in improving clinical outcomes in burn patients: a systematic review and meta-analysis of randomized controlled trials

**DOI:** 10.3389/fmed.2025.1485474

**Published:** 2025-08-08

**Authors:** Jiaqi Lou, Ziyi Xiang, Xiaoyu Zhu, Jingyao Song, Neng Huang, Jiliang Li, Guoying Jin, Youfen Fan, Shengyong Cui

**Affiliations:** ^1^Burn Department, Ningbo No. 2 Hospital, Ningbo, Zhejiang, China; ^2^Health Science Center, Ningbo University, Ningbo, Zhejiang, China; ^3^Faculty of Medicine, Institute of Pathology, University of Bonn, Bonn, Germany; ^4^School of Mental Health, Wenzhou Medical University, Whenzhou, China

**Keywords:** burn patients, androgen analog, oxandrolone, meta analysis, mortality

## Abstract

**Background:**

This latest systematic review and meta-analysis aim to examine efficacy and safety of androgen analog oxandrolone in burn patients.

**Methods:**

Relevant articles were retrieved from Pubmed, Embase, Cochrane, Web of science, International Clinical Trials Registry Platform, China National Knowledge Infrastructure, VIP Database for Chinese Technical Periodicals, Wanfang database and China Biology Medicine disc. The output measures were the weight loss in catabolic phase, weight gain in recovery phase, lean body mass in recovery phas, operation times, healing time of donor area, length of hospital stay/total body surface area burned (LOS/TBSA%), length of hospital stay (LOS), side effects, infection and mortality. Data were pooled and expressed as relative risk (RR) and standardized mean difference (SMD) with a 95% confidence interval (CI).

**Results:**

19 studies were included in this systematic review and meta-analysis, with 779 patients who received oxandrolone (treatment group) and 1,227 patients who received standard care or placebo (control group). The pooled data from all included studies demonstrated that the treatment group has significantly reduced weight loss in catabolic phase (SMD = 1.86; 95% CI: −0.13–3.84; *p* < 0.001, I^2^ = 95.0%), operation times (SMD = −0.69; 95% CI: −1.84–0.46, *p* < 0.001, I^2^ = 96.8%), LOS/TBSA% (SMD = −1.07; 95% CI: −2.43–0.29, *p* < 0.001, I^2^ = 98.1%), LOS (SMD = −0.55; 95% CI: −1.32–0.22, *p* < 0.001, I^2^ = 97.3%) and increased weight gain (SMD = 0.58; 95% CI: −1.21–2.38, *p* < 0.001, I^2^ = 95.1%), as well as lean body mass in recovery phase (SMD = 1.30; 95% CI: −0.47–3.24, *p* < 0.001, I^2^ = 95.0%). There were no significant differences in healing time of donor area (SMD = −1.48; 95% CI: −2.18–0.77, *p* = 0.116, I^2^ = 53.7%), side effects (RR = 1.13; 95% CI: 0.68–1.87, *p* = 0.174, I^2^ = 28.4%) and infection (RR = 0.83; 95% CI: 0.67–1.02, *p* = 0.639, I^2^ = 0.0%) between the two groups, and it did not significantly reduce mortality (RR = 1.04, 95% CI: 0.47–2.32, *p* = 0.013).

**Conclusion:**

Our meta-analysis showed that oxandrolone supplements are beneficial for burn patients as they significantly reduce the weight loss in catabolic phase, operation times, LOS/TBSA%, LOS, mortality and increase weight gain and lean body mass in recovery phase. However, this intervention has minimal impact on healing time of donor area, side effects and infection.

## 1 Background

Severe burns represent a major global health burden, with an estimated 18 million cases annually worldwide, leading to 265,000 deaths and consuming substantial healthcare resources, particularly in low-income regions (1). Severe burns often lead to an upsurge in pro-inflammatory cytokines and stress hormones (2), resulting in a state of heightened metabolism. This metabolic state is characterized by increased protein catabolism and inadequate protein synthesis, leading to a negative nitrogen balance that impacts nutritional status and the wound healing process (3). Furthermore, it can compromise the body’s immunity and predispose individuals to infections (4). In burn patients, reduced levels of androgen are observed (5) due to varying degrees of degeneration and necrosis in Leydig cells following extensive burns (6). These changes manifest as decreased cytoplasmic lipid droplets, expansion of smooth endoplasmic reticulum, condensed mitochondria, and diminished Golgi complexes (7, 8). Testosterone concentration rapidly decreases in adult male burn patients post-injury, with more severe burns correlating with greater reductions of it (9).

Burns are a significant global health burden, affecting millions of individuals annually and resulting in substantial mortality and consumption of medical resources. Oxandrolone, a synthetic testosterone analog, exhibits promising potential for enhancing wound healing by promoting protein synthesis while attenuating protein catabolism (10, 11). Its administration has been associated with accelerated wound closure in trauma patients along with increased wound contractility and collagen deposition (12). Oxandrolone exerts its effects by binding to androgen receptors, thereby promoting protein synthesis and inhibiting protein breakdown. It also plays a role in regulating metabolism and immune function (11). Specifically, it can increase the synthesis of muscle proteins, reduce the degradation of muscle fibers, and modulate the immune response to reduce inflammation and promote wound healing (11, 12). Although some studies (13, 14) have demonstrated its efficacy, high-quality research on this topic remains limited without comprehensive evidence review or summary regarding its application specifically in burn cases thus far. Moreover, previous findings (15–17) indicate that high-dose oxandrolone usage may lead to abnormal liver enzymes and disruptions in lipid metabolism; however, clear evidence confirming any adverse effects of oxandrolone on burns is lacking at present necessitating further investigation into its safety profile. Therefore, this systematic review is aimed to assess the androgen analog oxandrolone in improving clinical outcomes in burn patients.

## 2 Methods

The research protocol, outcomes, and relevant items in this systematic review were reported in accordance with the Preferred Reporting Items for Systematic Reviews and Meta-analyses (PRISMA) Statement (18). The protocol for this meta-analysis has been registered with PROSPERO (https://www.crd.york.ac.uk/PROSPERO/view/CRD42024564282, CRD42024564282).

### 2.1 Data source and search strategy

Relevant articles were searched in Pubmed, Embase, Cochrane, Web of science, International Clinical Trials Registry Platform, China National Knowledge Infrastructureris, VIP Database for Chinese Technical Periodicals, Wanfang database and China Biology Medicine disc using subject headings and keywords containing “Burns,” “Trauma,” “Wounds and Injuries,” “Wounds,” “Gonadal Hormones,” “Gonadal Steroid Hormones,” “Estradiol Congeners,” “Equilenin,” “Equilin,” “Estradiol,” “Estriol,” “Estrogenic Steroids, Alkylated,” “Estrogens, Catechol,” “Estrogens, Conjugated (USP),” “Estrogens, Esterified (USP),” “Estrone,” “Progesterone Congeners,” “Pregnenolone,” “Progesterone,” “Testosterone Congeners,” “Anabolic Androgenic Steroids,” “Androstane-3,17-diol,” “Androstenediol,” “Androstenedione,” “Androsterone,” “Dehydroepiandrosterone,” “Dihydrotestosterone,” “Etiocholanolone,” “Nandrolone” and “Testosterone.” For example, the PubMed search strategy included: “(((((Burns[Title/Abstract]) OR (Wounds and Injuries[Title/Abstract])) OR (Wounds[Title/Abstract])) OR (Trauma[Title/Abstract])) OR (diabetic foot[Title/Abstract])) AND (((((((((((((((((((((((((((Gonadal Hormones[Title/Abstract]) OR (Gonadal Steroid Hormones[Title/Abstract])) OR (Estradiol Congeners[Title/Abstract])) OR (Equilenin[Title/Abstract])) OR (Equilin[Title/Abstract])) OR (Estradiol[Title/Abstract])) OR (Estriol[Title/Abstract])) OR (Estrogenic Steroids, Alkylated[Title/Abstract])) OR (Estrogens, Catechol[Title/Abstract])) OR (Estrogens, Conjugated (USP)) OR (Estrogens, Esterified (USP)) OR (Estrone[Title/Abstract])) OR (Progesterone Congeners[Title/Abstract])) OR (Pregnenolone[Title/Abstract])) OR (Progesterone[Title/Abstract])) OR (Testosterone Congeners[Title/Abstract])) OR (Anabolic Androgenic Steroids[Title/Abstract])) OR (Androstane-3,17-diol[Title/Abstract])) OR (Androstenediol[Title/Abstract])) OR (Androstenedione[Title/Abstract])) OR (Androsterone[Title/Abstract])) OR (Dehydroepiandrosterone[Title/Abstract])) OR (Dihydrotestosterone[Title/Abstract])) OR (Etiocholanolone[Title/Abstract])) OR (Nandrolone[Title/Abstract])) OR (Testosterone[Title/Abstract])) OR (Gonadal Hormones[MeSH Terms])).” Similar strategies were adapted for other databases (e.g., Embase, Cochrane) using database-specific syntax and controlled vocabulary (Supplementary Table 1). In addition, the references of the included studies and relevant review articles were screened to identify eligible clinical trials that were not found through the database searches. The identified articles were imported, stored, and managed by EndNote 20. Each search result was independently reviewed for eligibility by two authors (Ziyi Xiang, Jiaqi Lou), and any disagreement was resolved by the corresponding author (Shengyong Cui). The literature search for each database was conducted from inception to December 2023. The search results were updated every 3 months during the study period to ensure the inclusion of the most recent studies.

### 2.2 Research question (PICOS framework)

This systematic review addressed the following question: (1) Population: Burn patients of all ages; (2) Intervention: Administration of androgen analogs (e.g., oxandrolone) alone or in combination with standard care; (3) Comparator: Placebo, standard care, or non-oxandrolone treatments; (4) Outcomes: Weight loss in the catabolic phase, weight gain in the recovery phase, lean body mass, operation times, healing time of donor area, length of hospital stay (LOS), LOS/TBSA%, side effects, infection, and mortality; and (5) Study Design: Randomized controlled trials (RCTs) (Supplementary Table 2).

### 2.3 Eligibility criteria

Inclusion criteria: (1) Type of study: RCT; (2) Participants: Burn patients of all ages; (3) Intervention: Androgen analog, like oxandrolone, alone or in combination with other treatment compared with placebo or non- oxandrolone; and (4) Outcomes: The weight loss in catabolic phase, weight gain in recovery phase, lean body mass in recovery phas, operation times, healing time of donor area, length of hospital stay/total body surface area burned (LOS/TBSA%), length of hospital stay (LOS), side effects, infection and mortality.

Exclusion criteria: (1) Studies without clear inclusion and exclusion criteria; (2) Outcomes that had not been clearly stated; (3) Uncontrolled studies; and (4) Preclinical studies in animal models. If multiple articles reported the same or overlapping data, the article with the longer duration of the intervention or larger sample size was included in this study.

### 2.4 Data extraction and quality assessment

Two co-authors (Jiaqi Lou and Xiaoyu Zhu) independently conducted article selection through the initial screening of titles and abstracts, followed by a full-text review to assess eligibility and sufficiency of information. For every study adhering to the eligibility requirements, relevant data including study sources (author, publication year, and country), study population characteristics (sample size, study design, type of subjects, gender ratio, baseline mean age), treatment details (drug, dosage, duration of intervention), and outcomes (weight loss in catabolic phase, weight gain, lean body mass in recovery phase, number of operations, healing time of donor area, LOS/TBSA%, LOS, side effects, infection and mortality) were gathered using a standardized data extraction template (19). A pilot-tested extraction template was used by two independent reviewers (Jiaqi Lou, Xiaoyu Zhu) to ensure accuracy. Discrepancies were resolved via consensus or third-author arbitration (Youfen Fan).

The methodological quality of the included randomized controlled trials was evaluated using the Cochrane Collaboration’s risk of bias tool, outlined in version 5.0.1 of the Cochrane Handbook for Systematic Reviews of Interventions (20). The risk of bias for each parameter was classified as either low, high, or unclear, conforming to the Cochrane Handbook’s criteria for Systematic Reviews (21). Independent authors, Jiaqi Lou and Shengyong Cui, performed data abstraction and quality appraisal. Any differences were resolved through discussion and, if consensus was not reached, a third researcher, Youfen Fan, provided an arbitrative judgment. Data synthesis was conducted using Review Manager (RevMan) 5.4.

### 2.5 Statistical analysis

Three co-authors (Jiaqi Lou, Jingyao Song, and Guoying Jin) concurrently extracted data from full-text studies using a shared worksheet. Information retrieved from the included studies encompassed: first author, year of publication, duration of treatment, study design, inclusion and exclusion criteria, intervention details, number of subjects, and outcomes. A third author, Jiliang Li, employed a standardized method for scrutinizing the data for validity. The methodological quality assessment, outlined in Table 1, conformed to the Cochrane Reviewers’ Handbook guidelines (20). Risk ratios (RR) and 95% confidence intervals (CIs) reported discrete numerical variables. Due to varying methods used to assess identical outcomes, standardized mean difference (SMD) was used as the summary statistic in this meta-analysis (22). The I^2^ statistic was utilized to quantify heterogeneity, and forest plots were jointly generated and double-checked by two authors (Jiaqi Lou and Neng Huang). When I^2^ statistics were below 50%, pooled outcomes were deemed as having low statistical heterogeneity, whereas figures above 50% indicated high statistical heterogeneity. A fixed-effects model estimated each low heterogeneity analysis, while high heterogeneity results were assessed using a random-effects model (21). To analyze potential sources of heterogeneity, a leave-one-out sensitivity evaluation was conducted, sequentially omitting studies to identify the confounding randomized control trial(s). Publication bias was assessed using funnel plots, Egger’s regression test, and a sensitivity analysis, where *p*-values below 0.05 were deemed statistically significant (23). If there was a discrepancy in funnel plot asymmetry or the Egger’s regression test results, the “trim and fill” method was utilized to substitute missing studies and estimate the influence of publication bias on the observed pooled effect size (24). All statistical analyses were performed using StataSE 15.1. A *p* < 0.05 was considered statistically significant for all tests except for the heterogeneity test, in which case a *p* < 0.10 was used.

**Table 1:** Summary of details in the randomized controlled trials (RCTs) studies included.

Study (author/year)	Journal	Country	Patients	Design	Group	Dosage and administration	Numbers of patients	Male (*n*)	Female (*n*)	Mean age (SD, Year)	%TBSA	Outcomes
Demling 1997 (25)	J Trauma	America	Patients with deep partial-thickness burns (30 to 50% TBSA burned)	Single center, RCT	High protein, high calorie diet+high protein supplement (MET-Rx) + Oxandrolone	10 mg oxandrolone, po, bid+High protein, high calorie diet+high protein supplement	7	NA	NA	36 ± 9	47 ± 6	(1)(2)(4)(10)
					High protein, high calorie diet+high protein supplement (MET-Rx)	High protein, high calorie diet+high protein supplement	8	NA	NA	34 ± 8	45 ± 8	
Demling 2000 (26)	J Crit Care	America	Patients with burns (40 to 70% TBSA burned)	Single center, RCT	Oxandrolone	Orally at a dose of 20 mg/day in two divided doses of four 2.5 mg tablets, either through a feeding tube or by mouth	11	NA	NA	49 ± 13	54 ± 9	(2)(4)(8)(10)
					Placebo	Given in the same fashion. The composition of the oxandrolone pill is 2.5 mg of oxandrolone combined with 150 mg of lactulose monohydrate, corn starch, and methyl cellulose	9	NA	NA	44 ± 6	49 ± 7	
Demling 2001-1 (1) (27)	Burns	America	Young patients with deep partial-thickness burns (30 to 55% TBSA burned) entering the injury recovery phase	Single center, RCT	Optimum nutrition and Exercise+Oxandrolone	Given 10 mg Oxandrolone orally twice a day for the next 4 weeks or until pre-burn weight had been restored+Optimum nutrition and Exercise	13	NA	NA	34 ± 5	47 ± 10	(1)(2)(3)(4)(10)
					Optimum nutrition and Exercise alone	Optimum nutrition and Exercise	12	NA	NA			
Demling 2001-1 (2) (27)	Burns	America	Old patients with deep partial-thickness burns (30 to 55% TBSA burned) entering the injury recovery phase	Single center, RCT	Optimum nutrition and Exercise+Oxandrolone	Given 10 mg Oxandrolone orally twice a day for the next 4 weeks or until pre-burn weight had been restored+Optimum nutrition and Exercise	8	NA	NA	60 ± 5	36 ± 5	(1)(2)(3)(4)(10)
					Optimum nutrition and Exercise alone	Optimum nutrition and Exercise	7	NA	NA			
Demling 2001-2 (28)	Burns	America	> 50%TBSA, and > 25% TBSA burned needed skin grafting, or > 25%TBSA burned with concurrent factors (60 years of age or older, malnutrition or diabetes)	Single center, RCT	Testosterone analog (oxandrolone)	20 mg oxandrolone, po, qd	16	13	3	49 ± 15	56 ± 15	(2)(4)(8)(10)
						0.1 mg/kg i.m. for HGH	24	16	8	40 ± 13	39 ± 12	
Zhang 2002 (29)	Guangxi Med J	China	Burn patients over the age of 18 years and under the age of 50 years with 30–60% TBSA burned and 10% deep partial-thickness burns”	Single center, RCT	Androgens (testosterone propionate) group	Application 3 days a week, qd, 25 mg, with 4 weeks in a row	16	NA	NA			(8)(10)
					Control	Same dose of saline, im	16	NA	NA			
Demling 2003 (30)	Burns	America	Burn patients transferred to rehabilitation center	Single center, RCT	Oxandrolone+Nutrition-Exercise	20 mg oxandrolone, po, qd + Nutrition-exercise program	23	NA	NA	43 ± 15	40 ± 14	(1)(2)(3)(10)
					Nutrition-Exercise	Nutrition-exercise program	22			41 ± 14	37 ± 15	
Murphy 2004 (31)	Surgery	America	Children with > 40%TBSA burned and younger than 18 years of age	Single center, RCT	Oxandrolone	0.1 mg/kg body weight oxandrolone was administered, po, bid	42	27	15	8.5 ± 5	54 ± 16	(4)
					Placebo	0.1 mg/kg body weight placebo was administered, po, bid	42	29	13	8.2 ± 5	60 ± 15	
Thomas 2004 (32)	J Trauma	America	After entering burn rehabilitation wards, children with ≥ 40% TBSA	Single center, RCT	Oxandrolone	Oxandrosterone (0.1 mg/kg, po, bid) from the 5th day after burn to 1 year after burn	10	8	2	9 ± 1	62 ± 5	(4)(7)
					Placebo	Placebo (0.1 mg/kg, po, bid) from the 5th day after burn to 1 year after burn	11	9	2	7 ± 2	60 ± 4	
Przkora 2005 (33)	Ann Surg	America	Children with > 40%TBSA burned and ≤ 18 years of age	Single center, RCT	Oxandrolone	0.1 mg/kg oxandrolone (BTG Pharmaceuticals, Iselin, NJ), po, bid, for 12 months after burn from the date of hospital discharge	30	20	10	9 ± 5	56 ± 16	(4)
					Placebo	0.1 mg/kg placebo, po, bid, for 12 months after burn from the date of hospital discharge	31	20	11	9 ± 5	61 ± 16	
Wolf 2006 (34)	J Burn Care Res	America	Burn patients with 20 to 60%TBSA	Multiple center, RCT	Oxandrolone	Oxandrolone 10 mg, po, q12h	46			39 ± 2	35 ± 2	(4)(5)(7)(10)
					Placebo	Placebo 10 mg, po, q12h	35			40 ± 3	36 ± 2	
Jeschke 2007 (35)	Ann Surg	America	> 40% TBSA burned children under the age of eight, and need at least a surgical intervention	Single center, RCT	Standard burn care+Oxandrolone	Oxandrolone 0.1 mg/kg body weight was administered, po, q12h + Standard burn care	45	31	14	8 ± 0.7	58 ± 2	(5)(6)(9)(10)
					Standard burn care+Placebo	Placebo 0.1 mg/kg body weight was administered, po, q12h + Standard burn care	190	112	78	7.7 ± 0.4	55 ± 2	
Pham 2008 (36)	J Burn Care Res	America	≥20% TBSA burned without concomitant traumatic injury	Multiple center, RCT	Oxandrolone treated	Within 7 days of admission for a duration of at least 7 days	59	46	13	42.3 ± 14.1	42.9 ± 16.9	(4)(6)(7)(9)(10)
	.				Oxandrolone non-treated	Duration of less than 7 days	58	44	14	42.9 ± 18.1	46.3 ± 20.3	
Tuvdendorj 2011 (37)	Surgery	America	age ≤18 years, at admission ≥40% TBSA burned	Single center, RCT	Oxandrolone	0.1 mg/kg, po, bid, for 6 months after discharge	12	10	2	10.4 ± 1.4	53 ± 2	(3)(9)
					Control	placebo, for 6 months after discharge	10	4	6	7.4 ± 1.2	58 ± 5	
Porro 2012 (38)	J Am Coll Surg	America	Patients aged 0–18 years with >30% TBSA burned and requiring at least one surgical intervention	Single center, RCT	Oxandrolone	Started within 48 h of the first surgery and administered Oxandrolone orally at a dose of 0.1 mg/kg, bid, for one year	70	45	25	8 ± 5	54 ± 15	(6)(7)(9)(10)
					Control	Started within 48 h of the first surgery and administered placebo orally at a dose of 0.1 mg/kg, bid, for one year	152	99	53	8 ± 5	57 ± 15	
Herndon 2016 (1) (39)	Ann Surg	America	Age ranged from 0.5 to 18 years with ≥30% TBSA burned	Single center, RCT	Oxandrolone	0.1 mg/kg body weight was administered, po, q12h, at least for 1 year	67	43	24	6 ± 0.5	54 ± 2	(4)(6)(10)
					Control	NA	248	146	102	6 ± 0.2	52 ± 1	
Herndon 2016 (2) (39)	Ann Surg	America	Age ranged from 0.5 to 18 years with ≥30% TBSA burned	Single center, RCT	Oxandrolone+Propranolol	4.0 ± 0.2 mg/kg/day, at least for 1 week, the dose was set to reduce the heart rate by 15%	103	72	31	5 ± 0.4	51 ± 1	(4)(6)(10)
					Control	NA	248	146	102	6 ± 0.2	52 ± 1	
Reeves 2016 (40)	Shock	America	Age ≤ 18 years with ≥30% TBSA burned	Single center, RCT	Long-Term Oxandrolone	0.1 mg/kg oxandrolone, po, bid, for 2 years	35	27	8	8.58 ± 0.96	50.57 ± 2.68	(4)(6)(7)(9)
					Control	Placebo, po, bid, for 2 years	84	55	29	7.17 ± 0.56	56 ± 1.35	
Chao 2018 (41)	Med Sci Sports Exerc	America	Children aged 7 to 17 years with burns	Single center, RCT	Rehabilitative Exercise Training (RET) + Oxandrolone+ Propranolol (Oxprop)	Oxandrolone was administered at 0.1 mg/kg (BTG Pharmaceuticals, Iselin, NJ), bid, during the patients entire stay in hospital and throughout the exercise training program. Propranolol was administered at a dose of 0.33 mg/kg of body weight every 4 h (1.98 mg/kg)	20	16	4			(1)(3)
					RET with a Placeb	NA	22	16	6			
Herndon 2018 (42)	Ann Surg	America	Children between 6 months and 18 years of age with burns over 30% TBSA and requiring skin grafting	Single center, RCT	OxProp	Oxandrolone (BTG Pharmaceuticals, West Conshohocken, PA) was administered at a dose of 0.1 mg/kg every 12 h for a minimum of 1 year; propranolol (Roxane Laboratories, Columbus, OH) was administered at a dose of 4.0 ± 0.2 mg/kg/day for a minimum of 1 year	126	91	35	7.4 ± 0.5	50 ± 1	(10)
					Control	NA	226	142	84	7.1 ± 0.4	52 ± 1	
Ali 2022 (43)	Burns	Egypt	Burn patients receiving treatment in the burn unit	Single center, RCT	Nandrolone decanoate (ND) + Escharectomy and skin grafting	Nandrolone decanoate injection in dose of 0.5 mg/kg/3 weeks, deep IM repeated till recovery	20	20	0	20–40岁	2–40%TBSA	(2)(4)(5)
					Escharectomy and skin grafting	Not received nandrolone decanoate injection	20	20	0			

Serial number definition:(1) Weight loss in catabolic phase; (2) Weight gain in recovery phase; (3) Lean body mass in recovery phase; (4) Side effects (mild liver dysfunction or local tissue edema); (5) Infection; (6) Mortality; (7) Operation times; (8) Healing time of donor area; (9) LOS/TBSA (d/TBSA); (10) LOS. The age data conforming to the normal distribution is expressed as M ± SD. NA: Not available; TBSA: Total body surface area; LOS: Length of stay; RCT: Randomized controlled trial; OxProp: Oxandrolone+Propranolol; qd: Quaque die; bid: Bis in die; im: Intramuscular Injection; po: peros.

## 3 Results

### 3.1 Characteristics of included studies

**Figure 1:**
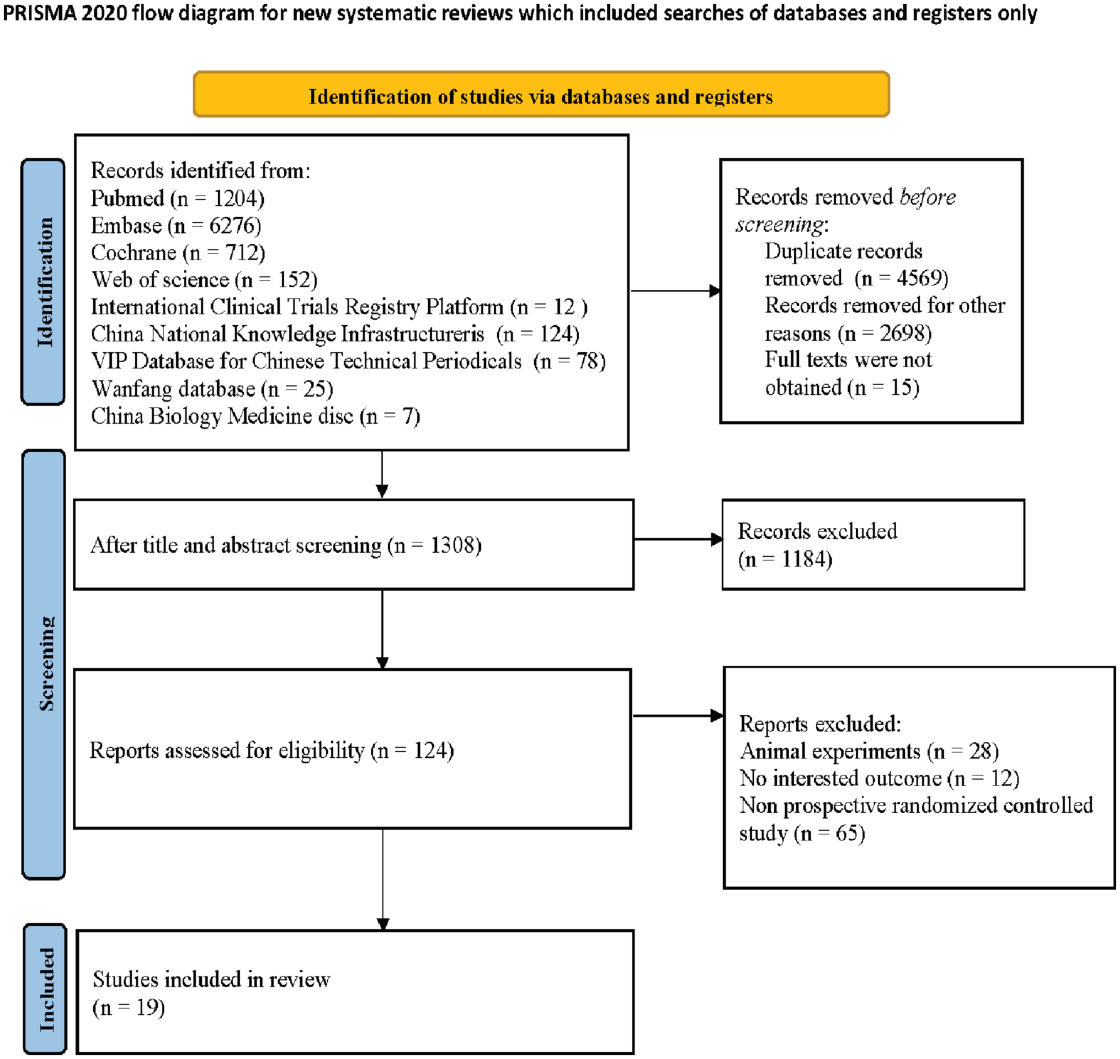
PRISMA diagram detailing the literature search and the study selection/exclusion process. PRISMA, preferred reporting items for systematic reviews and meta-analyses; RCT, randomized controlled trials.

As depicted by the PRISMA statement flowchart in Figure 1, the study selection process began with 8,590 records identified from the database search. Automated filtering eliminated 4,569 duplicates, leaving 4,021 records. Following the exclusion of 1,529 records not meeting the inclusion criteria and the manual removal of a further 1,184 duplicates, 124 records remained for review. Detailed scrutiny of these full articles resulted in 19 studies (25–43) meeting our inclusion criteria, thus qualifying for inclusion in this systematic review and meta-analysis. Table 1 presents the summarized attributes of these studies. The collective total of 2006 patients were classified into two groups: the androgen analog group (*n* = 779) and the control group (*n* = 1,227).

5 trials were explicit double-blinded except for the studies conducted by reference [25–29]. 2 studies were multi-center studies (34, 36), the others were single-center studies. These trials were published between 1997 and 2022, with sample sizes in individual trials ranging from 15 to 418. All trials were published in English except one (29) in Chinese. All trials were completed in the United States, except for the study by Zhang et al. (29), which was conducted in China, and the study by Ali et al. (43), which was conducted in Egypt.

The youngest participants were infants at gestational age of 6 months (29), and the vast majority were children participants (31–42). Interventions in the included trials consisted of different dosages of oxandrolone. All oxandrolone was administered orally in all trials, and the daily dose of oxandrolone in most trials was 10 or 20 mg, or calculated at 0.1 mg/kg. The androgen analog used in Ali et al.’s (43) trial was Nandrolone decanoate (ND), which was treated through injection at a dose of 0.5 mg/kg/3 weeks, combined with electrochemistry and skin grafting. The drug used by Zhang et al. (29) is testosterone propionate. In addition, in four studies (39–42), the experimental group received treatment with oxandrolone combined with propranolol. Chao et al. (41) and Demling et al. (30) combined physical exercise therapy on the basis of oxandrolone. The trial time of Pham et al. (36) was relatively shortest, with the minimum medication time for participants being only 1 week, while Reeves et al. (40) conducted the longest trial time, up to 2 years.

### 3.2 Risk of bias and quality assessment of individual studies

**Figure 2:**
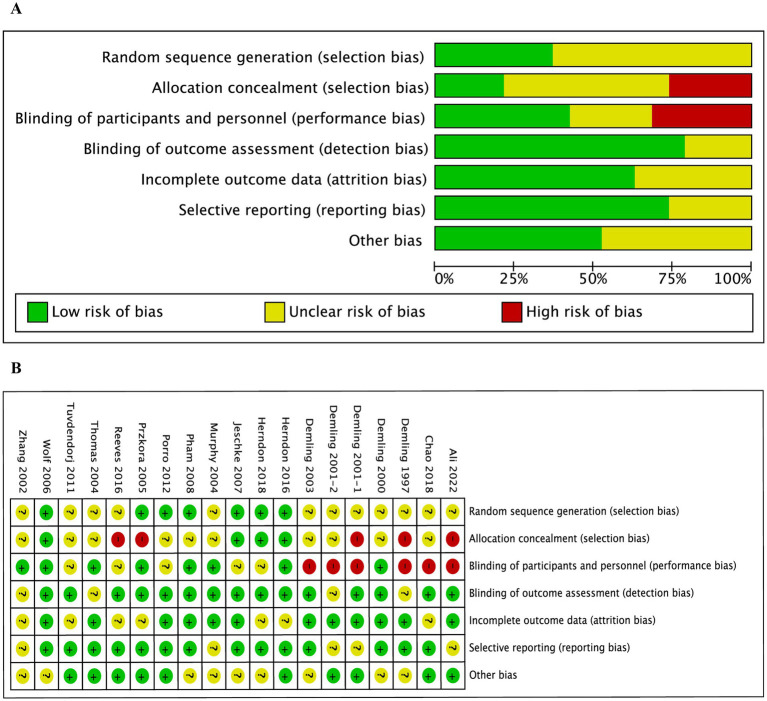
Risk of bias charts. **(A)** Risk of bias in the included studies; **(B)** Risk of bias summary for all included studies.

The Cochrane Collaboration’s risk of bias tool was employed to evaluate 19 studies. Figure 2 presents the risk of bias in the included trials across different quality domains of the risk of bias tool. Notably, five trials (25, 27, 33, 40, 43) did not adhere to allocation considerations, engendering high risk. Conversely, eight trials (26, 29, 31–34, 36, 39) displayed a clear double-blind design, while the remaining studies exhibited either low or unclear risks regarding the blinding of participants and key study personnel. Additionally, all the randomized controlled trials demonstrated a low or unclear risk of bias concerning incomplete outcome data and selective outcome reporting.

### 3.3 Meta-analysis findings

#### 3.3.1 Weight loss in catabolic phase

**Figure 3:**
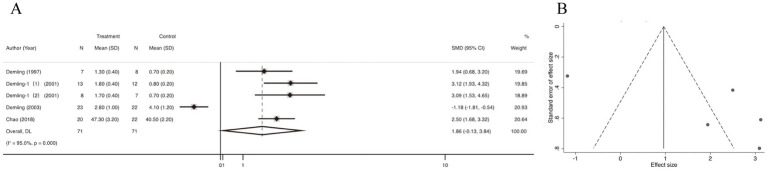
Forest plot and funnel plot of the meta-analysis illustrating the overall weighted effect size of androgen analog versus control on the weight loss in catabolic phase in burns. **(A)** Forest plot. The diamond symbol at the bottom of the forest plot represents the overall weighted estimate. **(B)** Funnel plot. The effect size “SMD” is shown on the abscissa, and the inverse of the standard error of the value of the effect size, SE (SMD), is shown on the ordinate. The dots in the figure are the individual studies included.

5 studies reported data on the weight loss in catabolic phase. Quantitative pooling of data revealed a significantly lower weight loss in catabolic phase in burn patients receiving oxandrolone compared with controls (SMD = 1.86; 95% CI: −0.13–3.84; *p* < 0.001, I^2^ = 95.0%) (Figure 3A). Publication bias was assessed using funnel plots, which appeared relatively asymmetrical, indicating significant publication bias (Figure 3B). Publication bias was assessed using the Egger’s regression test, which showed no possibility of statistically significant publication bias (*p* > 0.05) (Supplementary Figure 1A). We also performed publication bias assessment using funnel plots, which have slight asymmetrical distributions, indicating publication bias. Subsequently, trim-and-fill method was selected for adjustment, and the funnel plot and statistical results did not change, suggesting that our results was robust (Supplementary Figure 1B). In the leave-one-out sensitivity analysis, the removal of individual study did not lead to a significant change in the OR values (Supplementary Figure 1C).

#### 3.3.2 Weight gain in recovery phase

**Figure 4:**
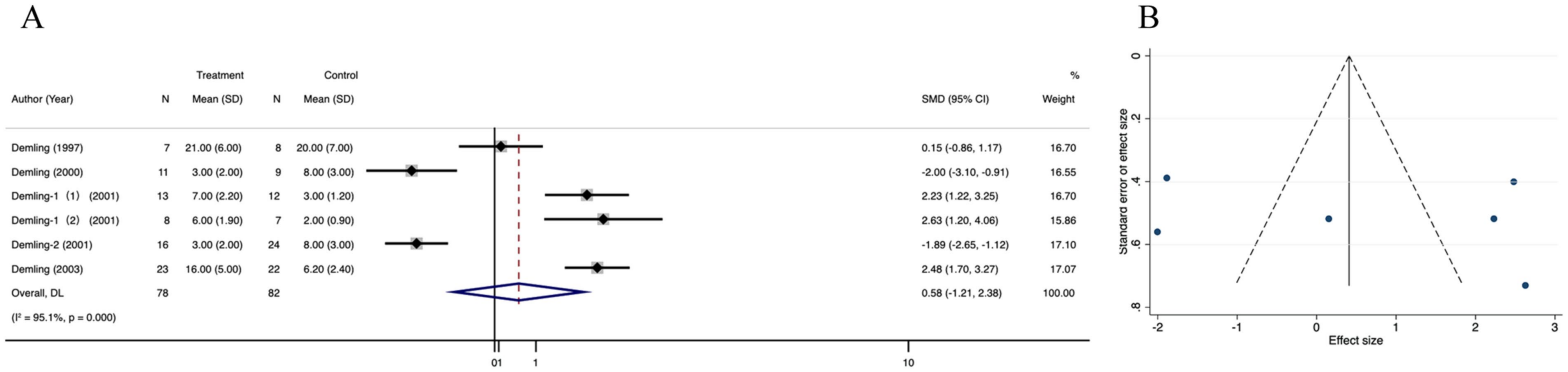
Forest plot and funnel plot of the meta-analysis illustrating the overall weighted effect size of androgen analog versus control on the weight gain in recovery phase in burns. **(A)** Forest plot. The diamond symbol at the bottom of the forest plot represents the overall weighted estimate. **(B)** Funnel plot. The effect size “SMD” is shown on the abscissa, and the inverse of the standard error of the value of the effect size, SE (SMD), is shown on the ordinate. The dots in the figure are the individual studies included.

Complete data on diarrhea were available in 6 studies. Pooled data from these trials demonstrated that there was no significant difference in the weight gain in recovery phase between the treatment and control groups (SMD = 0.58; 95% CI: −1.21–2.38, *p* < 0.001, I^2^ = 95.1%) (Figure 4A). The funnel plot showed no evidence of publication bias (Figure 4B), Egger’s regression test’ and trim-and-fill method’ results were not statistically different (*p* < 0.001) (Supplementary Figures 2A,B). Sensitivity analysis showed no significant change in the result after excluding each study (Supplementary Figure 2C).

#### 3.3.3 Lean body mass in recovery phase

**Figure 5:**
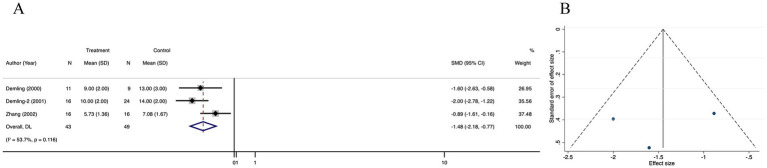
Forest plot and funnel plot of the meta-analysis illustrating the overall weighted effect size of androgen analog versus control on the lean body mass in recovery phas in burns. **(A)** Forest plot. The diamond symbol at the bottom of the forest plot represents the overall weighted estimate. **(B)** Funnel plot. The effect size “SMD” is shown on the abscissa, and the inverse of the standard error of the value of the effect size, SE (SMD), is shown on the ordinate. The dots in the figure are the individual studies included.

The lean body mass in recovery phase was not significantly different between the treatment and control groups in four trials (SMD = 1.30; 95% CI: −0.47–3.24, *p* < 0.001, I^2^ = 95.0%) (Figure 5A), The funnel plot (Figure 5B) and Egger’s test (Supplementary Figure 3A) showed no publication bias in these analyses (*p* for Egger’s test was 0.861). The corrected OR using the trim-and-fill method was 33.38% (95% CI, 24.20–35.15; random-effects model, *p* < 0.001) (Supplementary Figure 3B). There was no significant change in the pooled 95% CI upon removal of each study (Supplementary Figure 3C).

#### 3.3.4 Operation times

**Figure 6:**
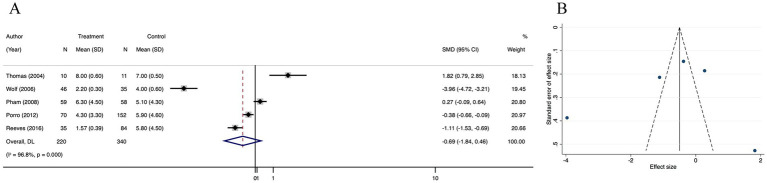
Forest plot and funnel plot of the meta-analysis illustrating the overall weighted effect size of androgen analog versus control on the operation times in burns. **(A)** Forest plot. The diamond symbol at the bottom of the forest plot represents the overall weighted estimate. **(B)** Funnel plot. The effect size “SMD” is shown on the abscissa, and the inverse of the standard error of the value of the effect size, SE (SMD), is shown on the ordinate. The dots in the figure are the individual studies included.

Meta-analysis of 5 trials showed that the operation times was significantly decreased in the treatment group than in the control group (SMD = −0.69; 95% CI: −1.84–0.46, *p* < 0.001, I^2^ = 96.8%) (Figure 6A). The I^2^ statistic indicated a high heterogeneity among the studies. However, funnel plot (Figure 6B) symmetry and the lack of significant difference in the Egger’s regression test (*p* = 0.483) (Supplementary Figure 4A) indicated that there was no detectable publication bias (Supplementary Figure 10). Through the results of the trim and fill analysis, it was identified that there was no distinct variation in the estimated value of the pooled effect size, indicating that the impact of publication bias was not evident and the outcomes were quite robust (Supplementary Figure 4B). A sensitivity analysis was conducted, revealing no impactful outliers (Supplementary Figure 4C).

#### 3.3.5 Healing time of donor area

**Figure 7:**
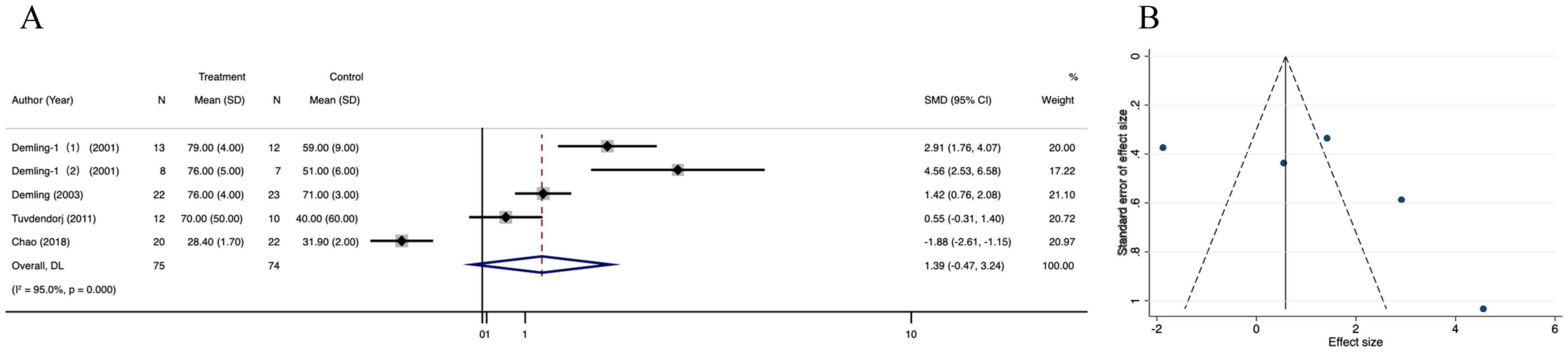
Forest plot and funnel plot of the meta-analysis illustrating the overall weighted effect size of androgen analog versus control on the healing time of donor area in burns. **(A)** Forest plot. The diamond symbol at the bottom of the forest plot represents the overall weighted estimate. **(B)** Funnel plot. The effect size “SMD” is shown on the abscissa, and the inverse of the standard error of the value of the effect size, SE (SMD), is shown on the ordinate. The dots in the figure are the individual studies included.

Data of healing time of donor area was reported in 3 studies. There was no significant difference in healing time of donor area between the treatment and control groups (SMD = −1.48; 95% CI: −2.18–0.77, *p* = 0.116, I^2^ = 53.7%) (Figure 7A). There was no significant publication bias in the selected studies, as indicated by funnel plot symmetry and the lack of significant difference in the Egger’s regression test (*p* = 0.472) (Figure 7A; Supplementary Figure 5A). Trim-and-fill method did not add any new studies to correct possible asymmetry in the funnel plot, and the estimates did not change (Supplementary Figure 5A). Sensitive analysis showed that no single study qualitatively altered the pooled healing time of donor area, providing evidence for the stability of the meta-analysis (Supplementary Figure 5C).

#### 3.3.6 Length of hospital stay/total body surface area burned

**Figure 8:**
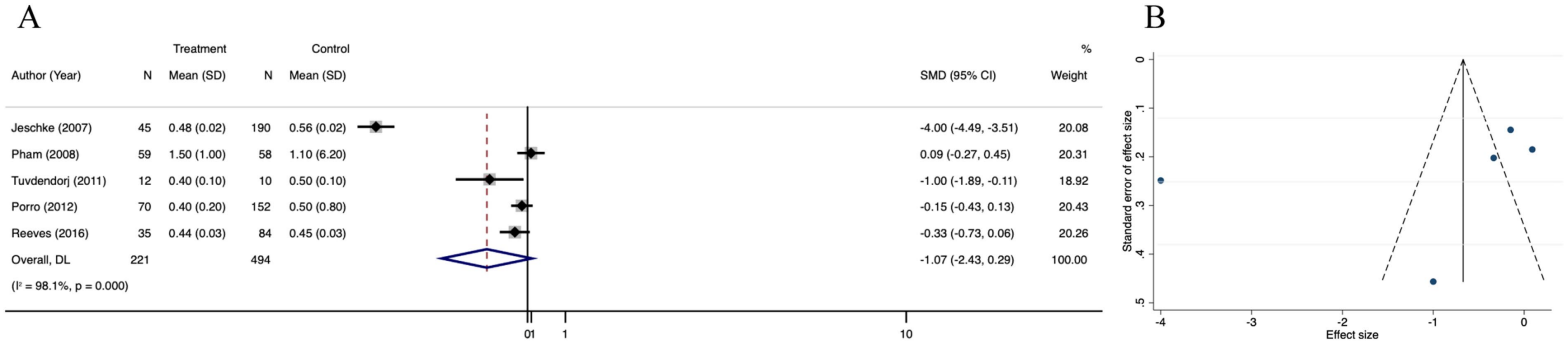
Forest plot and funnel plot of the meta-analysis illustrating the overall weighted effect size of androgen analog versus control on the length of hospital stay/total body surface area burned (LOS/TBSA%) in burns. **(A)** Forest plot. The diamond symbol at the bottom of the forest plot represents the overall weighted estimate. **(B)** Funnel plot. The effect size “SMD” is shown on the abscissa, and the inverse of the standard error of the value of the effect size, SE (SMD), is shown on the ordinate. The dots in the figure are the individual studies included.

Length of hospital stay/total body surface area burned (LOS/TBSA%) was significantly different between the treatment and control groups (SMD = −1.07; 95% CI: −2.43–0.29, *p* < 0.001, I^2^ = 98.1%) (Figure 8A). The funnel plot was partially symmetrical (Figure 8B), and Egger’s regression for funnel plot asymmetry revealed no risk of publication bias (*p* = 0.318) (Supplementary Figure 6A). Subsequently, we performed a trim and fill analysis, which not added any study, suggesting no existence of overlooked small study (Supplementary Figure 6B). Sensitivity analysis revealed no substantial change in LOS/TBSA% after omitting each of the 5 studies, confirming the stability of the results (Supplementary Figure 6C).

#### 3.3.7 Length of hospital stay

**Figure 9:**
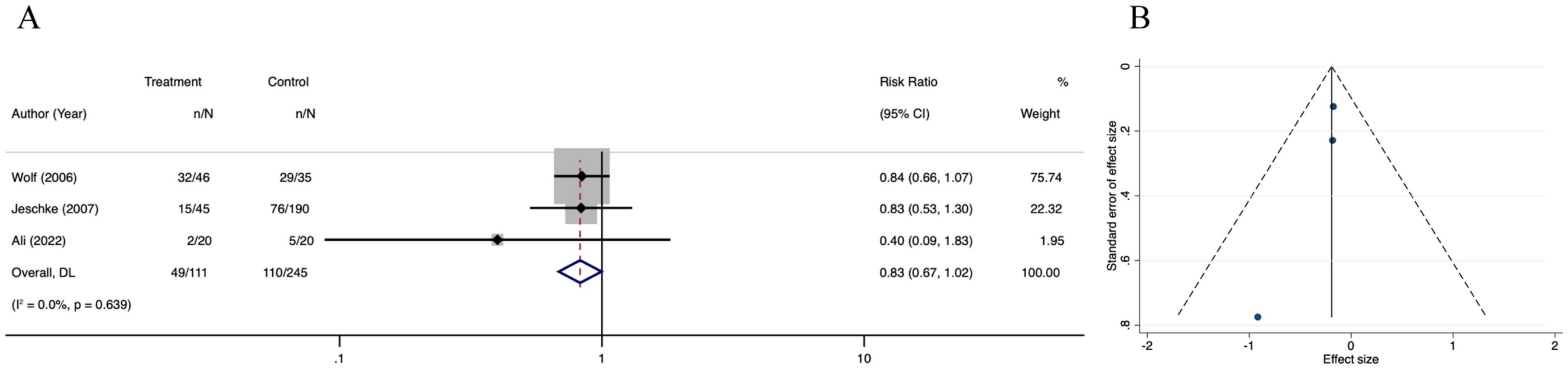
Forest plot and funnel plot of the meta-analysis illustrating the overall weighted effect size of androgen analog versus control on the length of hospital stay (LOS) in burns. **(A)** Forest plot. The diamond symbol at the bottom of the forest plot represents the overall weighted estimate. **(B)** Funnel plot. The effect size “SMD” is shown on the abscissa, and the inverse of the standard error of the value of the effect size, SE (SMD), is shown on the ordinate. The dots in the figure are the individual studies included.

Thirteen studies that reported the length of hospital stay (LOS) in the oxandrolone and control groups were included. Based on the random effects model, the oxandrolone treatment groups showed a significant reduction in LOS compared to the control groups (SMD = −0.55; 95% CI: −1.32–0.22, *p* < 0.001, I^2^ = 97.3%) (Figure 9A). Publication bias was checked using a funnel plot (Figure 9B), which was objectively verified using Egger’s regression test (*p* = 0.425) to confirm that there was no publication bias (Supplementary Figure 7A). The funnel plot appeared symmetric after trim-and-fill analysis of the linear estimator imputed (Supplementary Figure 7B). Sensitivity analysis revealed that the risk was not significantly altered by any of the individual studies (Supplementary Figure 7C).

#### 3.3.8 Side effects

**Figure 10:**
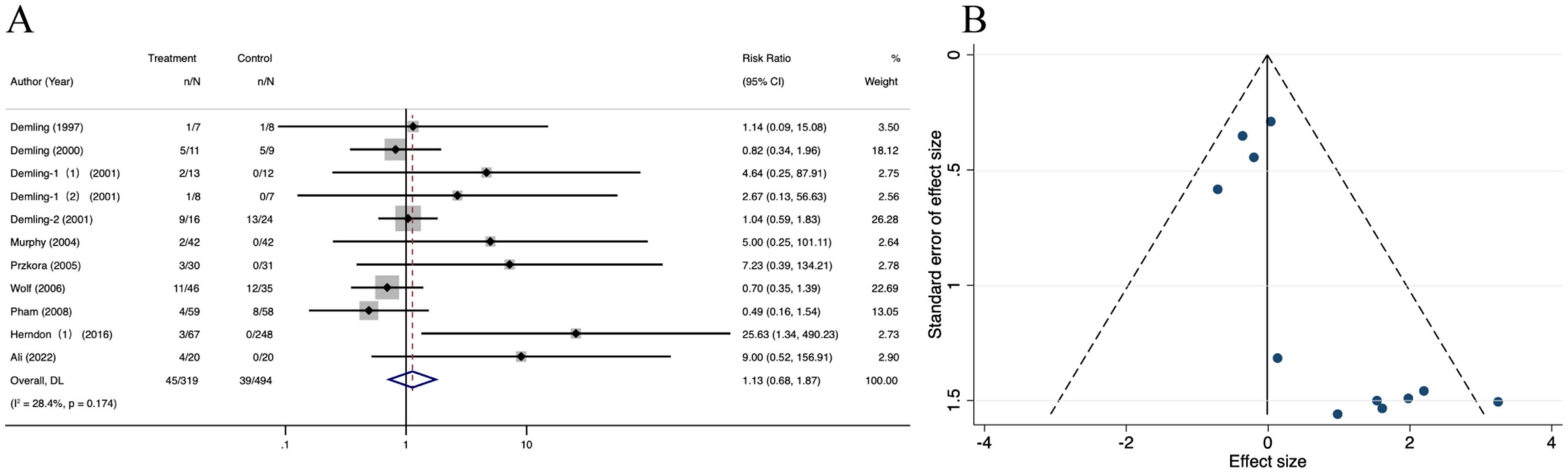
Forest plot and funnel plot of the meta-analysis illustrating the overall weighted effect size of androgen analog versus control on the side effects in burns. **(A)** Forest plot. The diamond symbol at the bottom of the forest plot represents the overall weighted estimate. **(B)** Funnel plot. The effect size “RR” is shown on the abscissa, and the inverse of the standard error of the value of the effect size, SE (SMD), is shown on the ordinate. The dots in the figure are the individual studies included.

There was no difference in side effects among the oxandrolone and control groups (RR = 1.13; 95% CI: 0.68–1.87, *p* = 0.174, I^2^ = 28.4%) (Figure 10A). Visual inspection of the funnel plot (Figure 10B) and further evaluation by Egger’s test (Supplementary Figure 8A) indicated no publication bias (*p* = 0.371). The trim-and-fill analysis (Supplementary Figure 8B) revealed a tiny pre- and post-combined effect size change, thereby indicating a small publication bias and more stable results. The sensitivity analysis results indicated that the meta-analysis results for side effects did not alter when each study was removed in turn and that the findings were robust (Supplementary Figure 8C).

#### 3.3.9 Infection

**Figure 11:**
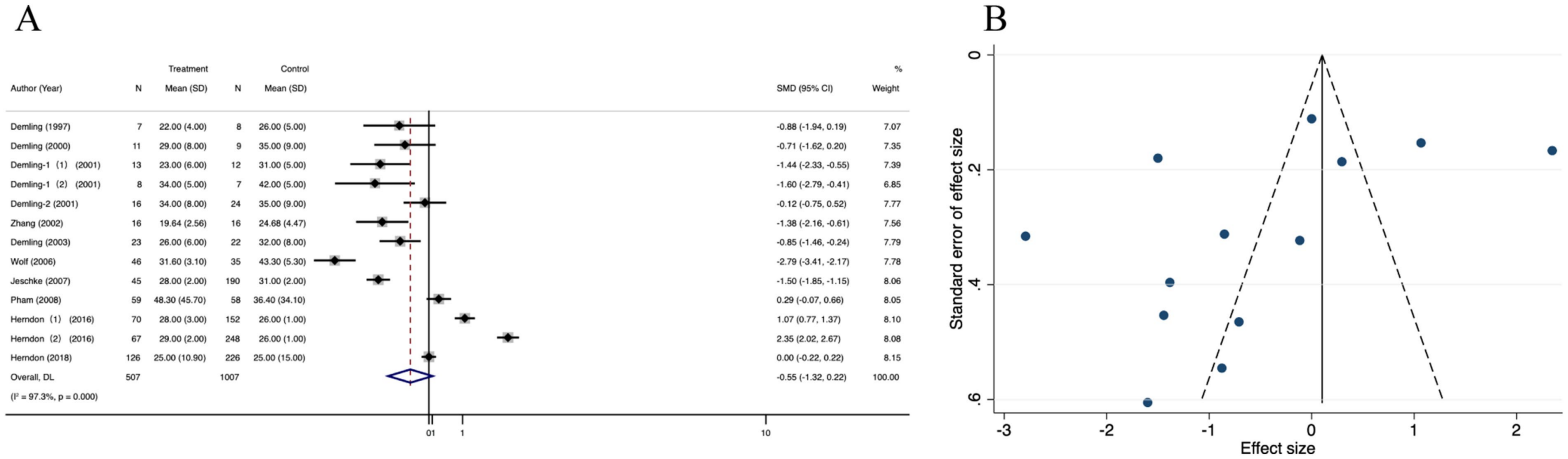
Forest plot and funnel plot of the meta-analysis illustrating the overall weighted effect size of androgen analog versus control on the infection in burns. **(A)** Forest plot. The diamond symbol at the bottom of the forest plot represents the overall weighted estimate. **(B)** Funnel plot. The effect size “RR” is shown on the abscissa, and the inverse of the standard error of the value of the effect size, SE (SMD), is shown on the ordinate. The dots in the figure are the individual studies included.

3 studies revealed no significant difference in infectious complications between the oxandrolone and control groups (RR = 0.83; 95% CI: 0.67–1.02, *p* = 0.639, I^2^ = 0.0%) (Figure 11A). A funnel plot (Figure 11B) showed that effect sizes were symmetrically distributed around the central dotted line. The results of Egger’s regression test and the trim and fill method indicated no publication bias (Supplementary Figures 9A,B). The leave-one-out sensitivity analysis showed that no single study significantly affected the pooled correlation from the meta-analysis, which indicated the reliability of the findings (Supplementary Figure 9C).

#### 3.3.10 Mortality

**Figure 12:**
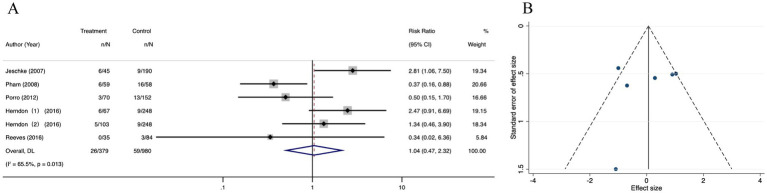
Forest plot and funnel plot of the meta-analysis illustrating the overall weighted effect size of androgen analog versus control on the mortality in burns. **(A)** Forest plot. The diamond symbol at the bottom of the forest plot represents the overall weighted estimate. **(B)** Funnel plot. The effect size “RR” is shown on the abscissa, and the inverse of the standard error of the value of the effect size, SE (SMD), is shown on the ordinate. The dots in the figure are the individual studies included.

Forest plot analysis showed no significant mortality reduction with oxandrolone (RR = 1.04; 95% CI: 0.47–2.32, *p* = 0.013, I^2^ = 65.5%). The CI crosses the null line (RR = 1), indicating no statistically significant effect, despite the nominal *p*-value (Figure 12A). Publication bias was suspected by observing the funnel plot (Figure 12B). The result from Egger’s regression test suggested that there were no publication bias (*p* = 0.628) (Supplementary Figure 10A). Then, we performed the trim-and-fill correction procedure, and the meta-analysis results did not alter. The findings were robust (Supplementary Figure 10B). The pooled results of sensitivity analysis were robust after omitting any of the studies once a time (Supplementary Figure 10C).

## 4 Discussion

This study presents a comprehensive systematic review and meta-analysis exploring the efficacy and safety of oxandrolone, representative of androgen analogs in burn treatment. This summary analysis suggests that oxandrolone can effectively mitigate weight loss during the catalytic phase, augment weight gain and lean body mass during the recovery phase, and lessen the number of surgeries and the length of hospital stays. Moreover, the incidence of patient infections or side effects, such as mild liver dysfunction and local tissue edema, parallels that of conventional treatments, indicating the clinical safety of oxandrolone.

Previous research (44) suggests that oxandrolone administration may reduce body mass loss, decrease nitrogen excretion, and expedite healing at donor sites in burn patients; however, these results were hampered by the limited number of included studies, significant publication bias, and inadequate research addressing pediatric patients. Li et al.’s meta-analysis (45), although focused solely on severely burned patients, included fewer participants than our study. Interestingly, despite their findings showing no significant reduction in mortality rate among burn patients receiving oxandrolone relative to the control group, our study suggests that oxandrolone does not affect the mortality during hospitalization. The significant *p*-value likely reflects heterogeneity (I^2^ = 65.5%) rather than true efficacy, necessitating re-evaluation of our initial conclusion. When comparing LOS reduction (SMD = −0.55) with Demling et al. (30) (SMD = −1.07), differences may stem from variations in patient age (adults vs. children) and oxandrolone dosing (20 mg/day vs. 0.1 mg/kg). In addition, safety concerns persist: Oxandrolone elevates hepatic transaminases via direct hepatotoxicity and disrupts lipid metabolism by reducing HDL-C. Long-term data (>5 years) on endocrine dysfunction (e.g., HPA axis suppression) remain scarce, warranting vigilance (15, 16).

Thermal injury can instigate complex alterations in metabolism and the immune system, including an often overlooked aspect: severe thermal injury’s significant impact on testicular function, thereby impairing spermatogenesis and testosterone secretion (46). Although no single drug can rectify the multiple defects in the endocrine and immune systems caused by severe burns (47), recent data suggest certain medications such as testosterone, oxandrolone, human recombinant growth hormone, insulin, metformin, and propranolol can enhance the nutritional status of severe burn patients. This includes factors like the net balance of skeletal muscle protein (48), providing effective assistance for treating burn patients in the hypermetabolic phase. Our study, among others, has focused on both the acute hypermetabolic phase of burn injury and the subsequent recovery phase, despite the absence of a clear demarcation between these periods. As one therapeutic strategy to mitigate the hypermetabolic response and associated insulin resistance post-burn, the ability of oxandrolone to increase the body’s protein synthesis may be linked to the upregulation of genes associated with transcription factors, growth factors, and muscle proteins, in addition to the downregulation of phosphatase I inhibitor (49). A previous DNA microarray analysis with oxandrolone revealed modifications in a large number of genes, significantly reducing the expression of various transcription factors and signaling molecules (50). This finding suggests that oxandrolone might also attenuate burn-related inflammation. However, opposing conclusions exist, with a placebo-controlled, randomized trial finding no beneficial impact of oxandrolone on the healing or closure of target pressure ulcers 8 weeks post-treatment (51). Another clinical trial of surgical patients requiring mechanical ventilation found those treated with oxandrolone necessitated a longer duration of ventilation support. This finding accentuates the potential exacerbating impact of oxandrolone on collagen deposition and fibrosis in later stages of acute respiratory distress syndrome (ARDS), thus prolonging the recovery phase for surgical patients (46). As severe burn patients often present complications like respiratory tract injuries or severe infections requiring mechanical ventilation, determining whether the net benefits of oxandrolone outweigh its potential respiratory harm merits further investigation.

Our study also observed oxandrolone in into the trial are almost as part of a complementary medicine. In the study by Demling et al. (25–28), oxandrolone was combined with high protein, high calorie diet, high protein supplement and/or exercise therapy, Herndon et al. (39) the study was applied in the intervention group oxandrolone joint propranolol treatment, and research such as Chao (41) has combined the advantages of the test in the test group, provides patients with a high protein diet plus exercise + oxandrolone joint naphthalene, comprehensive intervention measures. The other included studies also provided basic burn care protocols for patients, Most of these solutions is a surgical wound repair and care, is given priority to with enteral and parenteral nutrition therapy of nutritional support scheme, physical exercise therapy of comprehensive strategy (47), combined with some drug metabolism after more beneficial to the optimization of muscle mass burn patients, And help them to better restore physical function (48). In addition, there are differences in the dose of oxandrolone administered, and almost always in tablet form (49). In the future, more attention should be paid to the best dosage form of androgen analog in drug research and development to meet the universality and personalization of androgen analog in clinical practice.

This study offers a comprehensive review and analysis of high-quality RCTs, establishing the potential for oxandrolone to improve certain clinical outcomes in patients with burns, while manifesting a moderate safety profile. Nonetheless, the interpretations of the findings should be handled with caution due to the limitations, which include a small sample size, discrepancies in the data extracted, and variance in oxandrolone intervention modalities and doses (50). Future research should deliberate on the microscopic mechanisms underlying the effects of oxandrolone (or drugs with a similar structure) on metabolism and immune function in patients with burns at specific post-injury periods. Such research would provide insights on whether the promising effects observed extend to substantial clinical benefits, as well as elucidating the specific pharmacokinetics of oxandrolone in burn patients, aiding in determining the optimal administration method. Ultimately, the results from this study posit oxandrolone as a potentially valuable addition to existing burn treatments. Nonetheless, applicability to demographics with diverse burn conditions warrants further exploration.

## 5 Conclusion

Androgen analog oxandrolone supplementation plays a beneficial role in burn patients and presents a novel approach to the management of burns. This systematic review and meta-analysis supports the potential role of oxandrolone in reducing the weight loss in catabolic phase and operation times, as well as the LOS/TBSA%, LOS, and it also can increase weight gain in recovery phase and lean body mass in recovery phase in burn patients.

## Data Availability

The original contributions presented in the study are included in the article/Supplementary material, further inquiries can be directed to the corresponding author.

## Supplementary material

SUPPLEMENTARY FIGURE 1 The results of Egger’s regression test, trim and fill method and sensitivity analysis of the meta-analysis illustrating the overall weighted effect size of androgen analog versus control on the weight loss in catabolic phase in burns. **(A)** Result of Egger’s regression test. **(B)** Result of the trim and fill method. **(C)** Result of sensitivity analysis.

SUPPLEMENTARY FIGURE 2 The results of Egger’s regression test, trim and fill method and sensitivity analysis of the meta-analysis illustrating the overall weighted effect size of androgen analog versus control on the weight gain in recovery phase in burns. **(A)** Result of Egger’s regression test. **(B)** Result of the trim and fill method. **(C)** Result of sensitivity analysis.

SUPPLEMENTARY FIGURE 3 The results of Egger’s regression test, trim and fill method and sensitivity analysis of the meta-analysis illustrating the overall weighted effect size of androgen analog versus control on the lean body mass in recovery phase in burns. **(A)** Result of Egger’s regression test. **(B)** Result of the trim and fill method. **(C)** Result of sensitivity analysis.

SUPPLEMENTARY FIGURE 4 The results of Egger’s regression test, trim and fill method and sensitivity analysis of the meta-analysis illustrating the overall weighted effect size of androgen analog versus control on the operation times in burns. **(A)** Result of Egger’s regression test. **(B)** Result of the trim and fill method. **(C)** Result of sensitivity analysis.

SUPPLEMENTARY FIGURE 5 The results of Egger’s regression test, trim and fill method and sensitivity analysis of the meta-analysis illustrating the overall weighted effect size of androgen analog versus control on the healing time of donor area in burns. **(A)** Result of Egger’s regression test. **(B)** Result of the trim and fill method. **(C)** Result of sensitivity analysis.

SUPPLEMENTARY FIGURE 6 The results of Egger’s regression test, trim and fill method and sensitivity analysis of the meta-analysis illustrating the overall weighted effect size of androgen analog versus control on the length of hospital stay/total body surface area burned (LOS/TBSA%) in burns. **(A)** Result of Egger’s regression test. **(B)** Result of the trim and fill method. **(C)** Result of sensitivity analysis.

SUPPLEMENTARY FIGURE 7 The results of Egger’s regression test, trim and fill method and sensitivity analysis of the meta-analysis illustrating the overall weighted effect size of androgen analog versus control on the length of hospital stay (LOS) in burns. **(A)** Result of Egger’s regression test. **(B)** Result of the trim and fill method. **(C)** Result of sensitivity analysis.

SUPPLEMENTARY FIGURE 8 The results of Egger’s regression test, trim and fill method and sensitivity analysis of the meta-analysis illustrating the overall weighted effect size of androgen analog versus control on the side effects in burns. **(A)** Result of Egger’s regression test. **(B)** Result of the trim and fill method. **(C)** Result of sensitivity analysis.

SUPPLEMENTARY FIGURE 9 The results of Egger’s regression test, trim and fill method and sensitivity analysis of the meta-analysis illustrating the overall weighted effect size of androgen analog versus control on the infection in burns. **(A)** Result of Egger’s regression test. **(B)** Result of the trim and fill method. **(C)** Result of sensitivity analysis.

SUPPLEMENTARY FIGURE 10 The results of Egger’s regression test, trim and fill method and sensitivity analysis of the meta-analysis illustrating the overall weighted effect size of androgen analog versus control on the mortality in burns. **(A)** Result of Egger’s regression test. **(B)** Result of the trim and fill method. **(C)** Result of sensitivity analysis.
