# Supplementary figures and images for: The efficacy and safety of androgen analog oxandrolone in improving clinical outcomes in burn patients: a systematic review and meta-analysis of randomized controlled trials
Source: Front Med (Lausanne). 2025 Aug 8;12:1485474. doi: 10.3389/fmed.2025.1485474 (PMC12370634; doi:10.3389/fmed.2025.1485474)

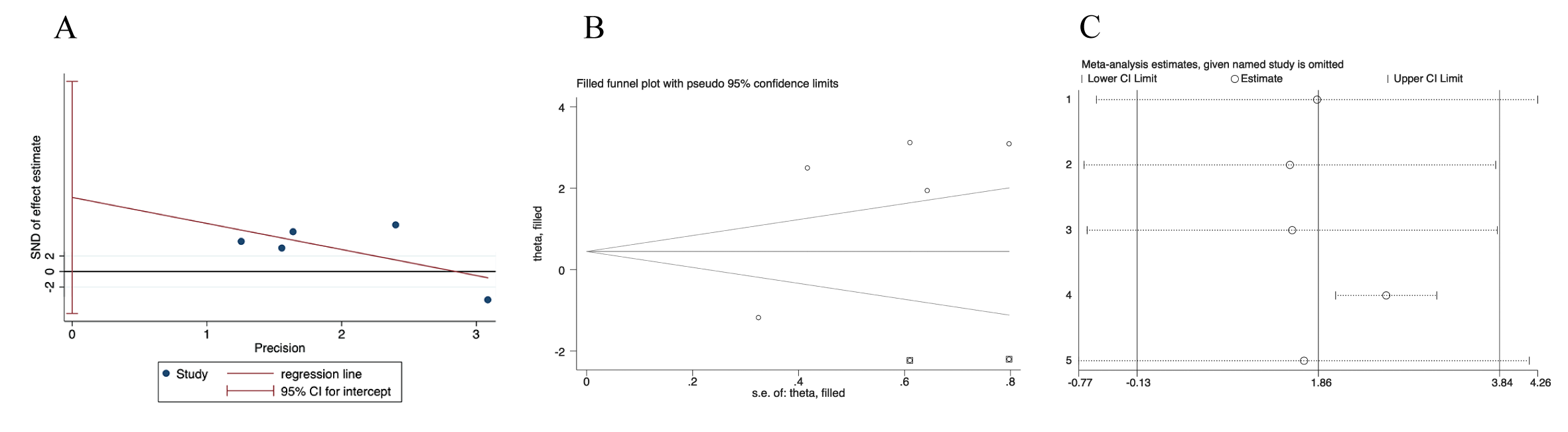

Supplement: SUPPLEMENTARY FIGURE 1 — The results of Egger’s regression test, trim and fill method and sensitivity analysis of the meta-analysis illustrating the overall weighted effect size of androgen analog versus control on the weight loss in catabolic phase in burns. (A) Result of Egger’s regression test. (B) Result of the trim and fill method. (C) Result of sensitivity analysis. [file Image_1.tif]

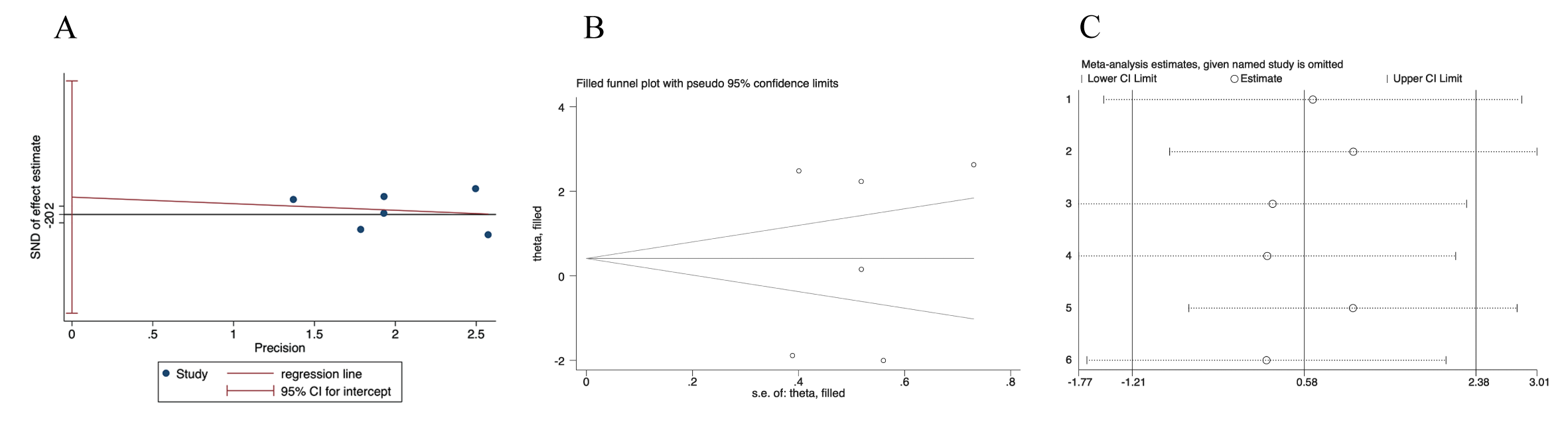

Supplement: SUPPLEMENTARY FIGURE 2 — The results of Egger’s regression test, trim and fill method and sensitivity analysis of the meta-analysis illustrating the overall weighted effect size of androgen analog versus control on the weight gain in recovery phase in burns. (A) Result of Egger’s regression test. (B) Result of the trim and fill method. (C) Result of sensitivity analysis. [file Image_2.tif]

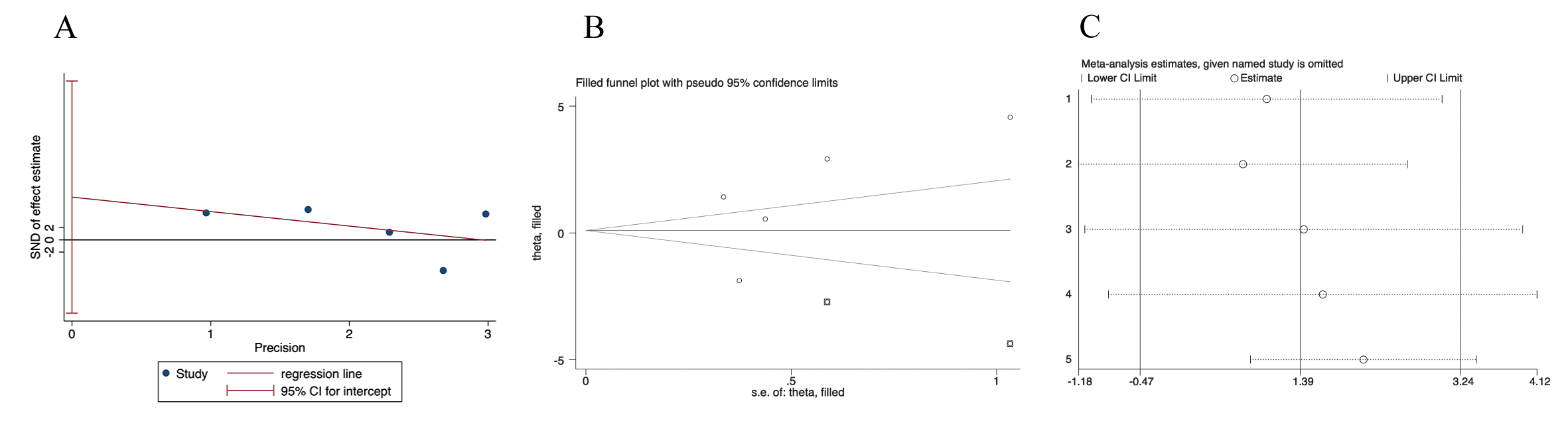

Supplement: SUPPLEMENTARY FIGURE 3 — The results of Egger’s regression test, trim and fill method and sensitivity analysis of the meta-analysis illustrating the overall weighted effect size of androgen analog versus control on the lean body mass in recovery phase in burns. (A) Result of Egger’s regression test. (B) Result of the trim and fill method. (C) Result of sensitivity analysis. [file Image_3.tif]

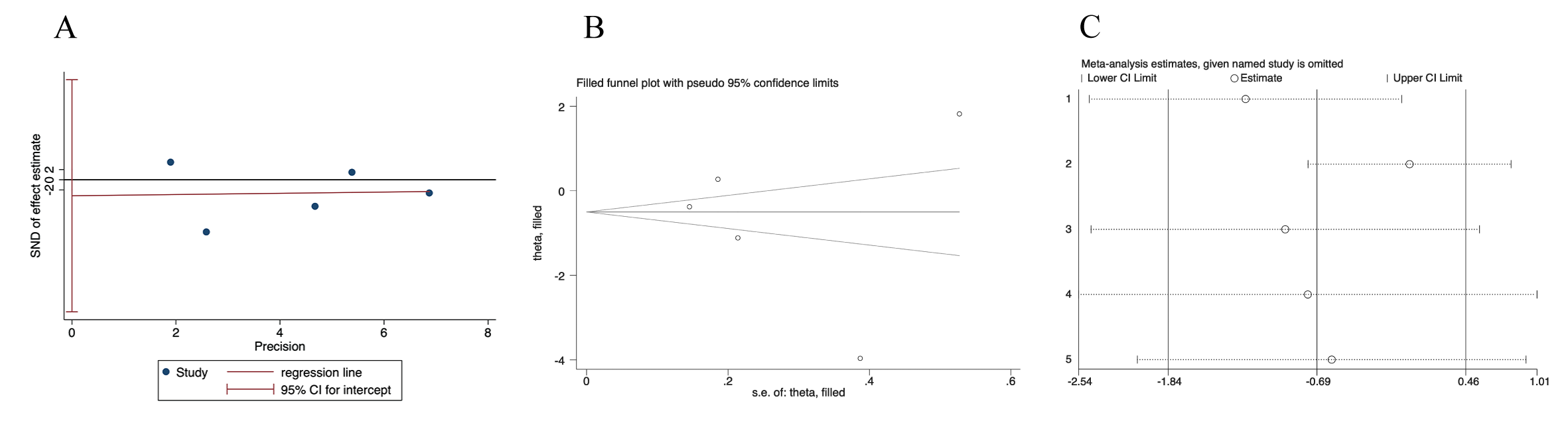

Supplement: SUPPLEMENTARY FIGURE 4 — The results of Egger’s regression test, trim and fill method and sensitivity analysis of the meta-analysis illustrating the overall weighted effect size of androgen analog versus control on the operation times in burns. (A) Result of Egger’s regression test. (B) Result of the trim and fill method. (C) Result of sensitivity analysis. [file Image_4.tif]

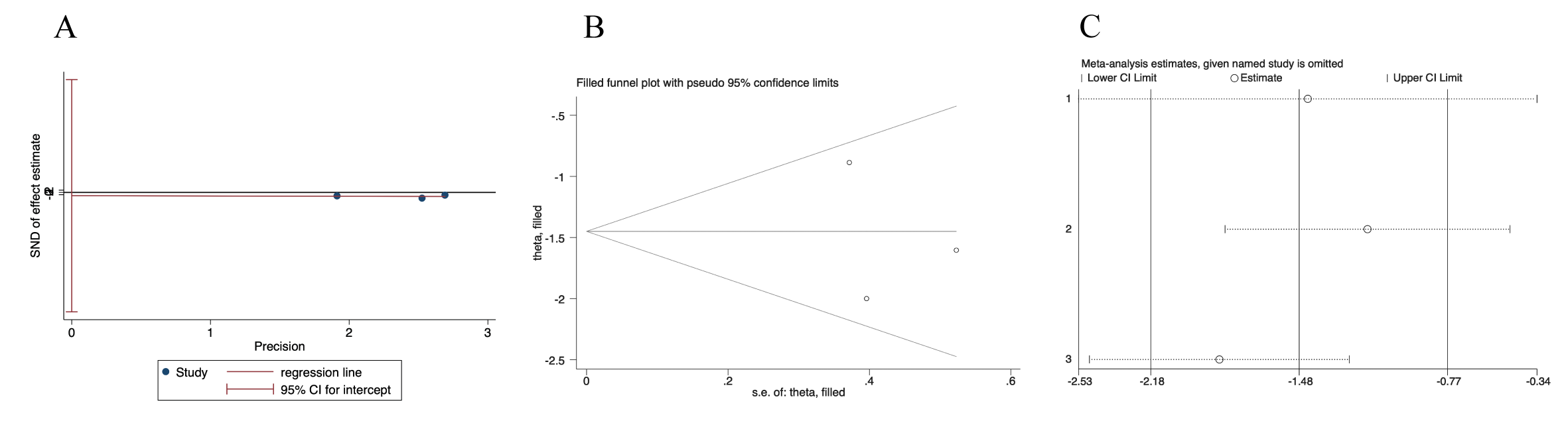

Supplement: SUPPLEMENTARY FIGURE 5 — The results of Egger’s regression test, trim and fill method and sensitivity analysis of the meta-analysis illustrating the overall weighted effect size of androgen analog versus control on the healing time of donor area in burns. (A) Result of Egger’s regression test. (B) Result of the trim and fill method. (C) Result of sensitivity analysis. [file Image_5.tif]

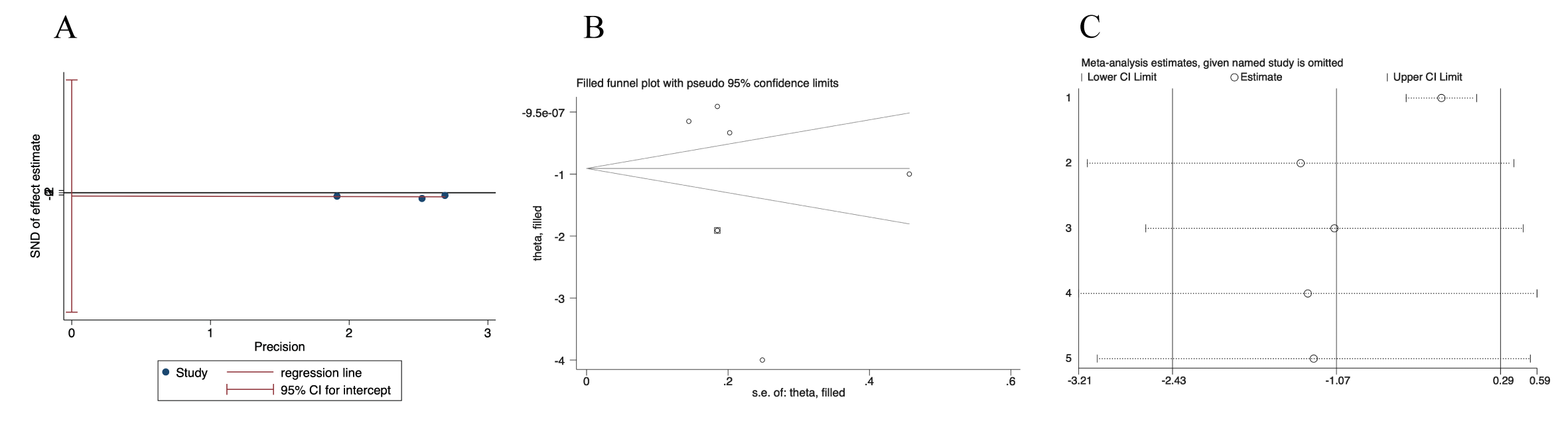

Supplement: SUPPLEMENTARY FIGURE 6 — The results of Egger’s regression test, trim and fill method and sensitivity analysis of the meta-analysis illustrating the overall weighted effect size of androgen analog versus control on the length of hospital stay/total body surface area burned (LOS/TBSA%) in burns. (A) Result of Egger’s regression test. (B) Result of the trim and fill method. (C) Result of sensitivity analysis. [file Image_6.tif]

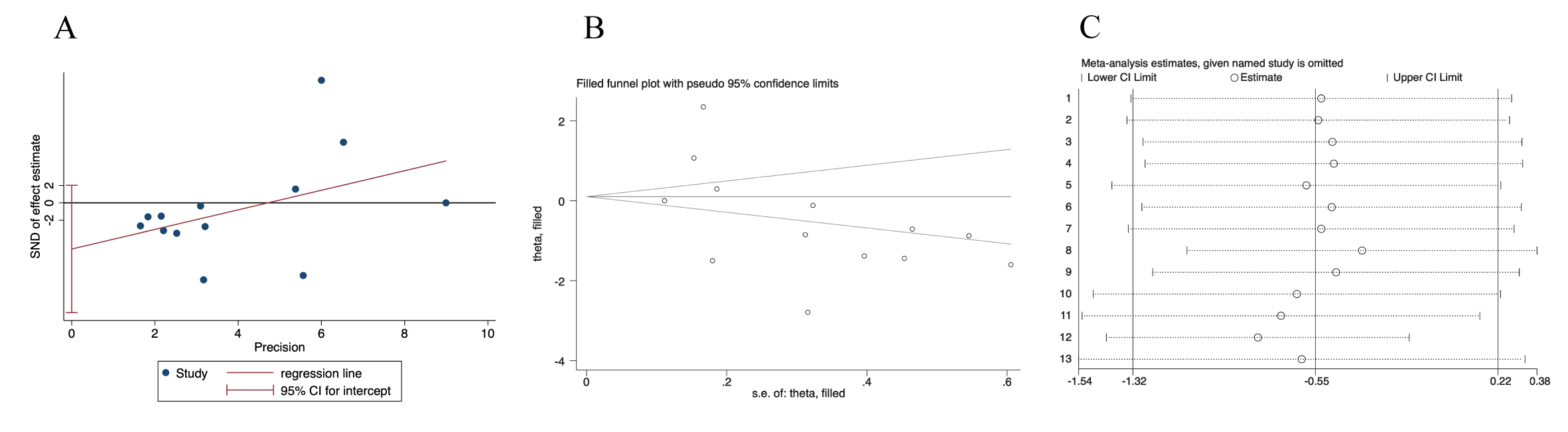

Supplement: SUPPLEMENTARY FIGURE 7 — The results of Egger’s regression test, trim and fill method and sensitivity analysis of the meta-analysis illustrating the overall weighted effect size of androgen analog versus control on the length of hospital stay (LOS) in burns. (A) Result of Egger’s regression test. (B) Result of the trim and fill method. (C) Result of sensitivity analysis. [file Image_7.tif]

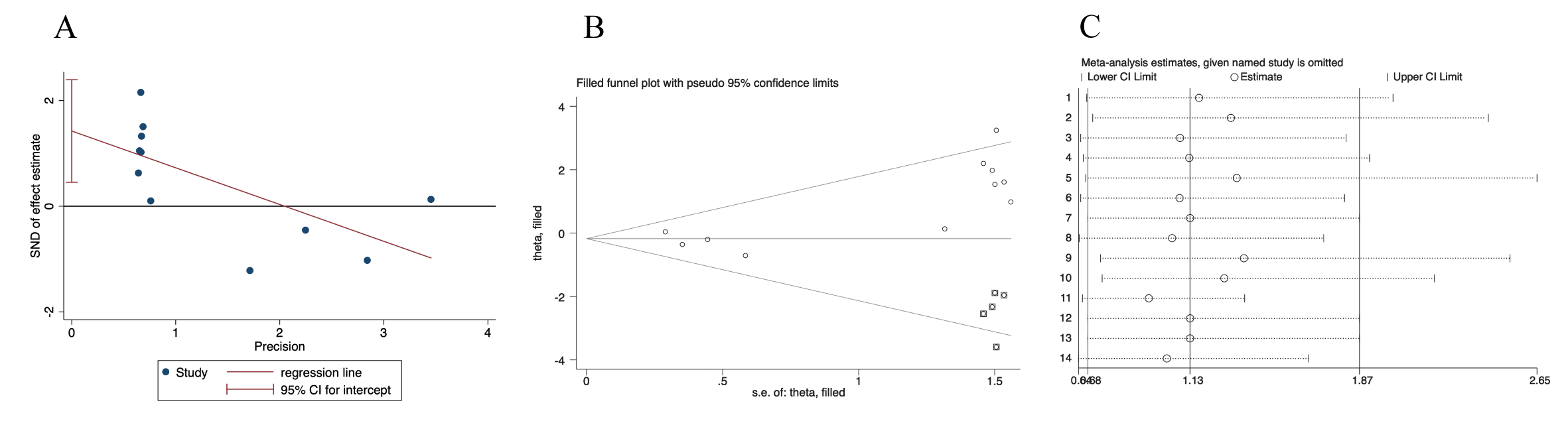

Supplement: SUPPLEMENTARY FIGURE 8 — The results of Egger’s regression test, trim and fill method and sensitivity analysis of the meta-analysis illustrating the overall weighted effect size of androgen analog versus control on the side effects in burns. (A) Result of Egger’s regression test. (B) Result of the trim and fill method. (C) Result of sensitivity analysis. [file Image_8.tif]

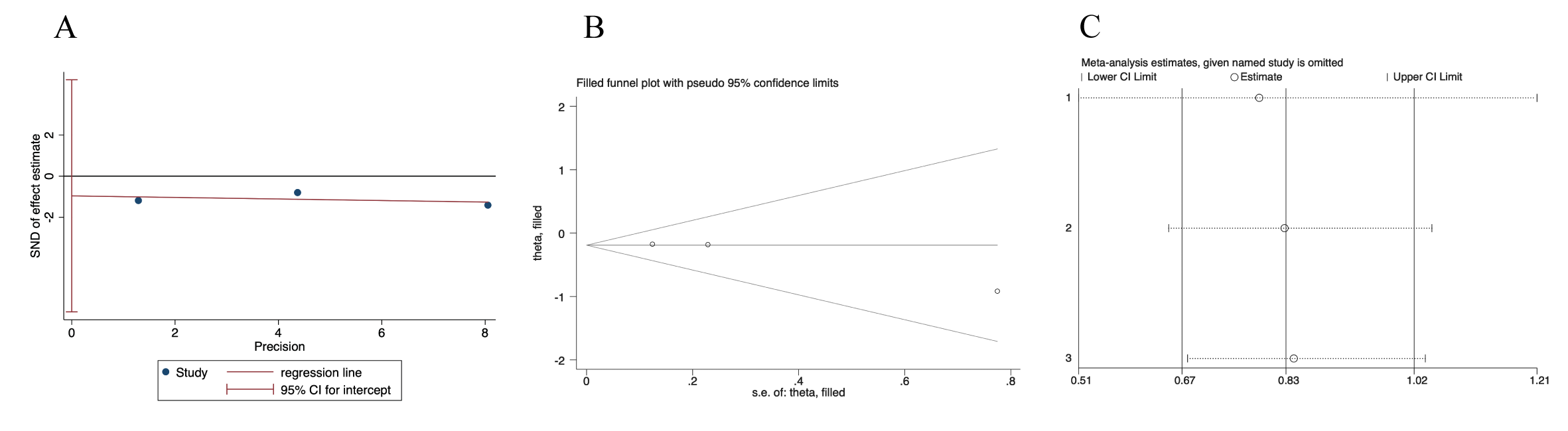

Supplement: SUPPLEMENTARY FIGURE 9 — The results of Egger’s regression test, trim and fill method and sensitivity analysis of the meta-analysis illustrating the overall weighted effect size of androgen analog versus control on the infection in burns. (A) Result of Egger’s regression test. (B) Result of the trim and fill method. (C) Result of sensitivity analysis. [file Image_9.tif]

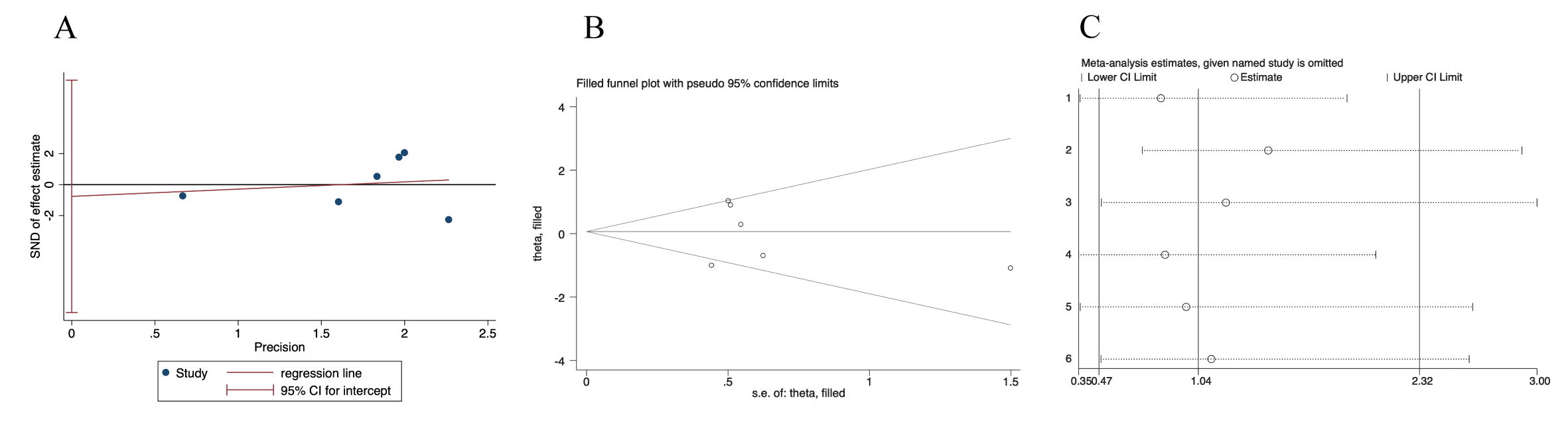

Supplement: SUPPLEMENTARY FIGURE 10 — The results of Egger’s regression test, trim and fill method and sensitivity analysis of the meta-analysis illustrating the overall weighted effect size of androgen analog versus control on the mortality in burns. (A) Result of Egger’s regression test. (B) Result of the trim and fill method. (C) Result of sensitivity analysis. [file Image_10.tif]
