# Supplementary material for: The efficacy and safety of androgen analog oxandrolone in improving clinical outcomes in burn patients: a systematic review and meta-analysis of randomized controlled trials
Source: Front Med (Lausanne). 2025 Aug 8;12:1485474. doi: 10.3389/fmed.2025.1485474 (PMC12370634; doi:10.3389/fmed.2025.1485474)
Supplement: Supplementary file 11 [file Table_1.docx]

**Supplementary Table 1.** Complete search query for other databases.

| Category | Description |
| --- | --- |
| Population | Burn patients of all ages |
| Intervention | Oxandrolone or other androgen analogs, alone or combined with other therapies |
| Comparator | Placebo, standard care, or non-oxandrolone treatments |
| Outcomes | Weight loss, weight gain, lean body mass, surgeries, LOS, mortality, etc. |
| Study Design | Randomized controlled trials (RCTs) |
