# Supplementary material for: The efficacy and safety of androgen analog oxandrolone in improving clinical outcomes in burn patients: a systematic review and meta-analysis of randomized controlled trials
Source: Front Med (Lausanne). 2025 Aug 8;12:1485474. doi: 10.3389/fmed.2025.1485474 (PMC12370634; doi:10.3389/fmed.2025.1485474)
Supplement: Supplementary file 12 [file Table_2.docx]

| **Supplementary Table 2.** PICOS Criteria for Study Inclusion. | |
| --- | --- |
| Database | Retrieval formulas for database |
| Embase | **Topic keyword search:** 'Burn' :de OR 'Burns' :de OR 'Burn Patient' :de OR 'Burn Patients' :de AND 'Oxandrolone' :de OR 'Anabolic Agent' :de OR 'Anabolic Steroid' :de AND 'Weight Loss' :de OR 'Recovery Phase' :de OR 'Recovery Period' :de AND 'Randomized Controlled Trial' :de OR 'Controlled Clinical Trial' :de**Keyword search:** ("Burn patient*" OR "Burn injured") AND ("Oxandrolone" OR "Anabolic steroid*" OR "Anabolic agent*") AND ("Weight loss" OR "Recovery phase" OR "Recovery period") AND ("Clinical trial*" OR "Randomized controlled trial*") |
| Cochrane Library | **Topic keyword search:** MeSH: "Burns" OR "Burn Injuries" OR "Burn Patients" AND "Oxandrolone" OR "Anabolic Agents" AND "Weight Loss" OR "Recovery Phase" AND "Randomized Controlled Trials"**Keyword search:** ("Burn patients" OR "Burn injuries") AND ("Oxandrolone" OR "Anabolic steroids") AND ("Weight loss" OR "Recovery phase") AND ("Randomized controlled trials" OR "RCTs") |
| Web of Science | **Topic keyword search:** TS=("Burn patients" OR "Burn injuries") AND TS=("Oxandrolone" OR "Anabolic steroids") AND TS=("Weight loss" OR "Recovery phase") AND TS=("Randomized controlled trials" OR "Clinical trials")**Search for fields such as author and journal:** Fields such as AU (author), SO (journal name), PY (year) can be used to limit specific requirements. |
| International Clinical Trials Registry Platform | Search by entering the following keyword combinations in the search box: (Burn patients OR Burn injuries) AND Oxandrolone AND (Weight loss OR Recovery phase) AND (Randomized controlled trials OR Clinical trials)。 |
| China National Knowledge Infrastructure (CNKI) | **Advanced search:** Select the "Topic" field, enter "Burn Patient", and select the "And" relationship; Select the "Theme" field again, enter "Oxygen Dragon", and choose the "And" relationship; Next, select the "Theme" field, enter "Weight Loss" or "Recovery Period", and choose the "And" relationship; Finally, select the "Literature Type" field and enter "Randomized Controlled Trial".**Precise search:** Double quotation marks can be used for precise search, such as TS=("burn patient" AND "oxyandrolone" AND ("weight loss" or "recovery period") AND DT=("randomized controlled trial"). |
| VIP Database for Chinese Technical Periodicals | **Keyword search: Enter "burn patient" and "oxyandrolone" and "weight loss" or "recovery period" in the "Chinese title, keywords, abstract" field.** **Classification number search: First search for classification numbers related to burns, endocrinology, etc., and then combine keywords for search.** |
| Wanfang Database | **Advanced Search: Select the "Topic" field, enter "Burn Patient", and select the "And" relationship; Select the "Theme" field again, enter "Oxygen Dragon", and choose the "And" relationship; Next, select the "Theme" field, enter "Weight Loss" or "Recovery Period", and choose the "And" relationship; Finally, select the "Literature Type" field and enter "Randomized Controlled Trial".**  **Multi field combination search: Enter "randomized controlled trial of Oxandrolone for weight loss and recovery in burn patients" in the "Full text" field, and use double quotes, spaces, etc. appropriately to accurately match.** |
| China Biology Medicine disc | Enter "Burn Patient AND Oxandrolone AND (Weight Loss OR Recovery Period)" in the "Chinese Title, Keywords, Abstract" field, or enter the corresponding keywords in the "Topic" field for search. |
